# RNA-seq reveals the circular RNA and miRNA expression profile of peripheral blood mononuclear cells in patients with rheumatoid arthritis

**DOI:** 10.1042/BSR20193160

**Published:** 2020-04-03

**Authors:** Jianting Wen, Jian Liu, Pingheng Zhang, Hui Jiang, Ling Xin, Lei Wan, Yue Sun, Dan Huang, Yanqiu Sun, Yan Long, Ying Zhang, Bingxi Bao, Guanghan Sun

**Affiliations:** 1 Anhui University of Traditional Chinese Medicine, Heifei 230031, Anhui Province, China; 2 Department of Rheumatology and Immunology, First Affiliated Hospital of Anhui University of Traditional Chinese Medicine, Heifei 230038, Anhui Province, China; 3 Institute of Rheumatology, Anhui Academy of Traditional Chinese Medicine, Heifei 230012, Anhui Province, China; 4 Zhujiang Hospital, Southern Medical University, Guangzhou 510280, Guangdong Province, China

**Keywords:** circular RNAs, miRNAs, qPCR, rheumatoid arthritis

## Abstract

**Objective:** Circular RNAs (circRNAs) are a significant class of molecules involved in a wide range of diverse biological functions that are abnormally expressed in many types of diseases. The present study aimed to determine the circRNAs specifically expressed in peripheral blood mononuclear cells (PBMCs) from rheumatoid arthritis (RA) patients to identify their possible molecular mechanisms.

**Methods:** To identify the circRNAs specifically expressed in RA, we started by sequencing the of PBMCs circRNA and microRNAs (miRNAs) from a RA group (*n* = 3) and a control group (*n* = 3). We constructed a network of differentially expressed circRNAs and miRNAs. Then, we selected differentially expressed circRNAs in PBMCs from 10 RA patients relative to 10 age- and sex-matched controls using real-time quantitative reverse transcription-polymerase chain reaction (RT-qPCR). Spearman’s correlation test was used to evaluate the correlation of circRNAs with biochemical measurements.

**Results:** A total of 165 circRNAs and 63 miRNAs were differently expressed between RA patients and healthy people according to RNA-seq, including 109 circRNAs that were significantly up-regulated and 56 circRNAs that were down-regulated among the RA patients. RT-qPCR validation demonstrated that the expression levels of hsa_circ_0001200, hsa_circ_0001566, hsa_circ_0003972, and hsa_circ_0008360 were consistent with the results from the sequencing analysis. Then, we found that there were significant correlations between the circRNAs and disease severity.

**Conclusion:** Generally, these results suggest that expression of hsa_circ_0001200, hsa_circ_0001566, hsa_circ_0003972, and hsa_circ_0008360 in PBMCs from RA patients may serve as potential biomarkers for the diagnosis of RA, and these circRNAs may influence the occurrence and development of RA.

## Introduction

Rheumatoid arthritis (RA) is a chronic disease characterized by autoimmunity and systemic inflammation [1]. The main pathological changes of RA are synovial inflammation, pannus formation and joint destruction caused by an imbalance of cytokines [2,3]. RA mainly occurs in people aged 20–50 years and affects approximately 1% of the world’s population, and the incidence rate among females is 2–3 times that of males [4]. If not diagnosed early and treated, RA will eventually lead to joint deformity and loss of function and seriously affect the quality of life of RA patients. Although some great advances in the treatment of RA have been made in recent years, such as the biologic therapies, it still cannot be cured, may be because our understanding of the pathogenesis of RA is still very limited [5]. Thus, it is necessary to study the genetic and molecular abnormalities of RA by screening for new biomarkers.

CircRNAs are an important class of widespread endogenous noncoding RNAs that may play important roles in diseases by regulating gene expression processes, such as transcription, splicing and translation [6,7]. Recently, there is accumulating evidence that circRNAs are abundant in mammals and function as molecular sponges for their target miRNAs to regulate gene expression. circRNAs can efficiently bind and prevent miRNAs transcription, further influencing downstream mRNA expression and thus participating in various diseases [8,9]. An increasing body of evidence has shown that several circRNAs play an important role in the regulation of autoimmune disease, leading to aberrant gene expression that may contribute to the progression of autoimmune diseases including RA, SLE, etc. [10,11]. Although circRNAs are known to be significant biomolecules for understanding gene regulation and the pathogenesis of related diseases, very little research has been conducted as to whether these circRNAs can also be used as biomarkers for both diagnosis and medical care in RA.

In this research, RNA-seq was used to identify circRNAs and miRNAs differentially expressed between RA and healthy people. We characterized the circRNAs expression profiles in RA patients by comparing three samples of peripheral blood mononuclear cells from a model group and three samples from healthy people and evaluated the expression of the identified circRNAs using RT-qPCR. Finally, we conducted a correlation analysis between circRNAs and the RA disease activity indicators, DAS28 and SF-36 scores. Our results not only revealed the potential molecular mechanism of RA pathogenesis but also demonstrated that differentially expressed circRNAs may provide new molecular targets for the treatment of RA.

## Materials and methods

### Patient variables

A total of 10 RA patients who were hospitalized in the First Affiliated Hospital of Anhui University of Traditional Chinese Medicine from June 2019 to July 2019 were recruited for this research. Ten age- and sex-matched healthy subjects who underwent regular physical examination at the Department of Health from the same hospital were recruited as control subjects. Written informed consent was obtained from all of the recruited patients, and our research was approved by the First Affiliated Hospital of Anhui University of Traditional Chinese Medicine Ethical Committee. All RA patients fulfilled the new classification criteria of RA proposed by the American Rheumatology Society (ACR) and the European Union against Rheumatism (EULAR) in 2010 [12]. Patients who had serious heart, liver, or kidney diseases or a mental disorder, and pregnant or lactating women, were excluded.

### Biochemical measurements

Erythrocyte sedimentation rate (ESR), high-sensitivity C-reactive protein (CRP), rheumatoid factor (RF), anti-cyclic citrullinated peptide antibody (CCP), immunoglobulin A (IGA), immunoglobulin G (IGG), immunoglobulin M (IGM), complement 3 (C3) and complement 4 (C4) were measured enzymatically using an autoanalyzer (Hitachi 747; Hitachi, Tokyo, Japan). The Short Form-36 Health Survey (SF-36), Disease Activity Score (DAS28), Visual Analogy Scale (VAS), Anxiety Self-Assessment Scale (SAS), and Depression Self-Assessment Scale (SDS) scores were based on a questionnaire survey of the patients.

### PBMCs preparation and total RNA extraction

PBMCs were immediately separated after the collection of blood samples from each donor according to the following protocol. Five milliliters of blood diluted in 5 ml saline solution was layered on 4 ml Ficoll-Paque PLUS (GE Healthcare, Uppsala, Sweden). After centrifugation for 20 min at 2000 rpm, the intermediate floc was collected by washing twice with the same volume of saline solution. The intermediate floc was gathered by centrifuging twice for 5 min at 1000 rpm. The PBMCs were then frozen at –80°C. Then, total RNA was extracted from the PBMCs using TRIzol reagent according to the manufacturer’s instructions.

### RNA-seq analysis

Total RNA was extracted from the PBMCs using TRIzol reagent (Life Technologies/Thermo Fisher Scientific, Waltham, MA, U.S.A.) according to the manufacturer’s instructions. First, ribosomal RNA was removed from the total RNA using the Epicentre Ribo-Zero rRNA Removal kit (Illumina, San Diego, CA, U.S.A.). The ribosome-depleted RNA was then treated with RNase R (Epicentre) and fragmented to approximately 200 bp. Then, the purified RNA was subjected to first strand cDNA synthesis according to the instructions provided by the NEBNext superdirected RNA library kit (NEB, Beverly, MA, U.S.A.). The cDNA fragments were repaired using an End-It DNA End Repair kit, modified by the Klenow fragment to add an A to the 3′ end of the DNA fragments, and finally ligated to adapters. Purified first-strand cDNA was then subjected to 13–15 cycles of PCR amplification. The library products were evaluated using the Agilent 2200 TapeStation and Qubit 2.0 (Life Technologies, Carlsbad, CA, U.S.A.) and then diluted to 10 pM for cluster generation in situ on the HiSeq3000 pair-end flow cell followed by sequencing (2×150 bp) on HiSeq3000 (Illumina). We used the Skewer software to dynamically remove the sequence fragments and low quality fragments from the 3′end of the sequencing data. Also, we used the FastQC software to analyze the quality of the preprocessed data and calculate the base ratio of Q20 and Q30. BWA software is used to compare the preprocessed data to rRNA sequence database in turn. The reference genome version is GRCh38(hg38).

### Quantitative RT-qPCR analysis

PBMCs from 10 RA patients and 10 healthy controls were used for the validation of six circRNAs by real-time qPCR. cDNA was synthesized from total RNA using a Prime-Script RT reagent kit with gDNA Eraser (TaKaRa, Shiga, Japan). GAPDH was used as an internal control. The data were analyzed using the 2∧-∆∆Ct method and are presented as relative expression levels from three independent experiments.

### Statistical analysis

All data are expressed as the mean ± standard deviation (mean ± SD). The groups were compared to evaluate their statistical significance using the Mann–Whitney test, Student’s *t*-test, Wilcoxon signed-rank test or chi-square test, as appropriate. Associations between parameters were analyzed using the Spearman rank correlation. *P* < 0.05 was considered as statistical significance. The threshold value we used to screen differentially expressed circRNAs and miRNAs was a fold change ≥ 2.0 (*P* < 0.05). All statistical analyses were performed using SPSS (version 22.0 SPSS, Chicago, IL, U.S.A.).

## Results

### Clinical and biochemical features of the included individuals

As shown in Table 1, there was no obvious difference between the RA patients and healthy people in terms of sex and age. The two groups baselines are consistent and comparable.

**Table 1:** Clinical characteristics of study population

Index	RA	Control	*P*
Sex (M/F)	1/9	1/9	1.000
Age (years)	44.4 ± 10.73	43.9 ± 10.49	0.922
Silk time (years)	3.23 ± 3.10	NA	NA
ESR (mm/h)	41.40 ± 26.51	NA	NA
Hs-CRP (mg/l)	23.34 ± 35.96	NA	NA
RF (U/ml)	112.49 ± 79.77	NA	NA
CCP (U/ml)	92.30 ± 67.91	NA	NA
IGA (g/l)	2.64 ± 0.60	NA	NA
IGG (g/l)	13.78 ± 3.43	NA	NA
IGM (g/l)	1.61 ± 0.56	NA	NA
C3 (g/l)	1.33 ± 0.24	NA	NA
C4 (g/l)	0.33 ± 0.24	NA	NA
DAS28 score	6.77 ± 0.93	NA	NA
VAS score	6.94 ± 0.94	NA	NA
SAS score	56.43 ± 4.87	NA	NA
SDS score	56.35 ± 5.77	NA	NA
SF-36 score	1302.1 ± 14.99	NA	NA

### Identification of the differentially expressed circRNAs and miRNAs in the RA and healthy participants

**Figure 1:**
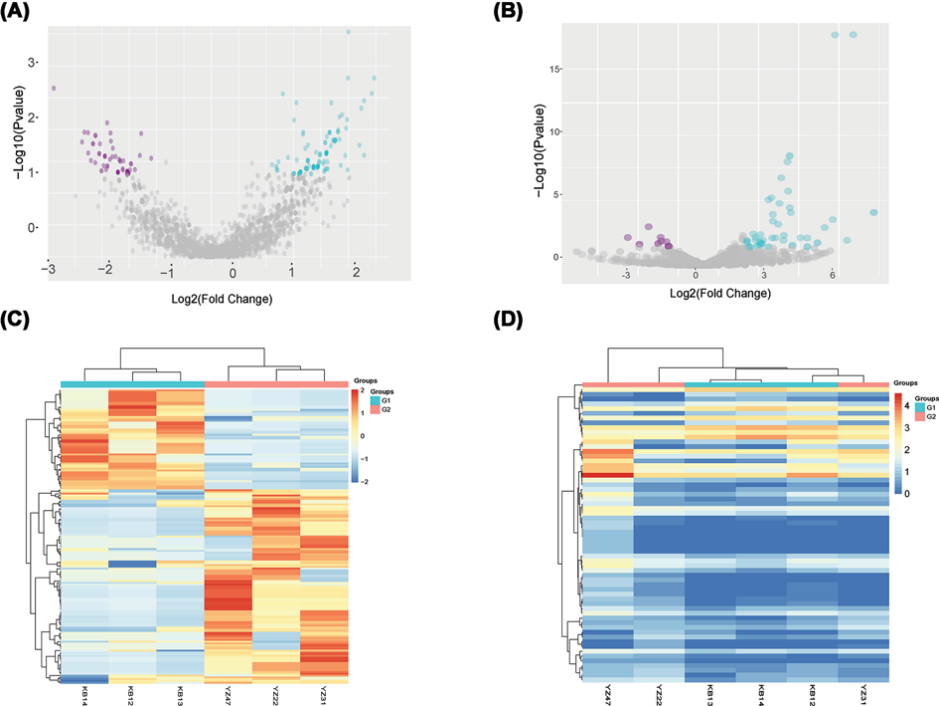
Sequences determining the circRNAs and miRNAs expression profiles in three RA patients and three healthy controls (**A**) CircRNAs (red point) in the volcano plots represent the 1.5-fold up- and down-regulated logarithmized circRNAs with statistical significance (*P*<0.05). (**B**) MiRNAs (red point) in the volcano plots represent the 1.5-fold up- and down-regulated logarithmized miRNAs with statistical significance (*P*<0.05). (**C**) Heat map of differentially expressed circRNAs. Columns represent samples, and rows represent each circRNA. Red indicates high relative expression, and blue indicates low relative expression. (**D**) Heat map of differentially expressed miRNAs. Columns represent samples, and rows represent each miRNA. Red indicates high relative expression, and green indicates low relative expression.

To identify the circRNAs and miRNAs that were differentially expressed in RA, we performed sequencing analysis of the circRNAs and miRNAs in the PBMCs from three patients with RA and three healthy people. After the raw data were normalized, the differentially expressed circRNAs and miRNAs were identified between the two groups. We identified 165 circRNAs (109 up-regulated and 56 down-regulated, Supplementary Table S1) and 63 miRNAs (51 up-regulated and 12 down-regulated, Supplementary Table S2) that were differentially expressed between the two populations by screening for log2-fold-changes greater than 1 and *P* < 0.05 (Figure 1A,B). A heat map was constructed to group the circRNAs and miRNAs based on their expression levels among the samples (Figure 1C,D).

### GO and pathway analysis of the differentially expressed circRNAs

**Figure 2:**
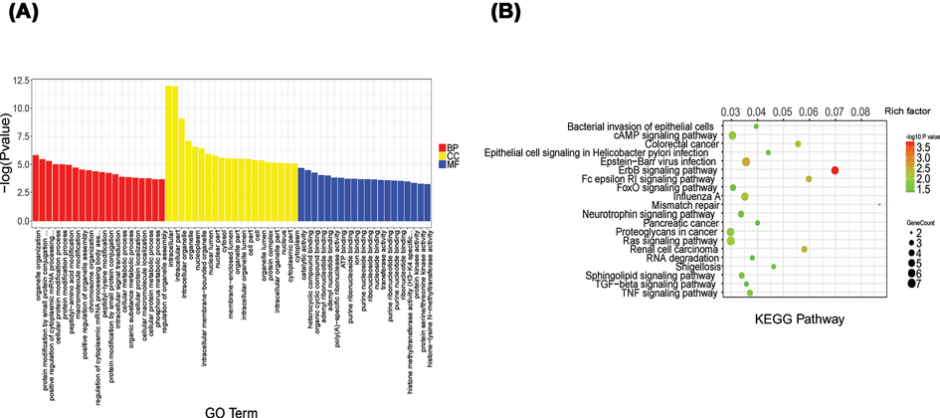
GO analysis and KEGG pathway annotation of differentially expressed circRNAs (**A**) The top 20 significantly obvious changes in GO biological process classification. Red, yellow and blue bars respectively represent biological process, cellular component, and molecular function, respectively. (**B**) The top 20 significant obvious changes in KEGG pathway classification. The bar plot shows the top Enrichment Score (−log10 (*P* value)) value of the significant pathway. The color represents the difference, darker indicating a greater difference. The circle represents the relationship between the gene and the pathway, the larger circles represent a stronger relationship between the gene and the pathway.

GO analysis was used to categorize and describe the biological functions of genes and gene products (Figure 2A). The ontology covered three domains: biological process, cellular component and molecular function. According to GO analysis, the genes were mainly involved in a variety of biological processes, such as organelle organization, protein modification process and cellular signal instruction; Cellular components, such as the nuclear lumen, nuclear part and cellular; and molecular function, such as catalytic activity, ATP binding and protein kinase activity. The KEGG pathway analysis showed that the TNF signaling pathway, TGF-β signaling pathway and the FoxO signaling pathway were the most significantly different (Figure 2B).

### Construction of a circRNA–miRNA co-expression network

**Figure 3:**
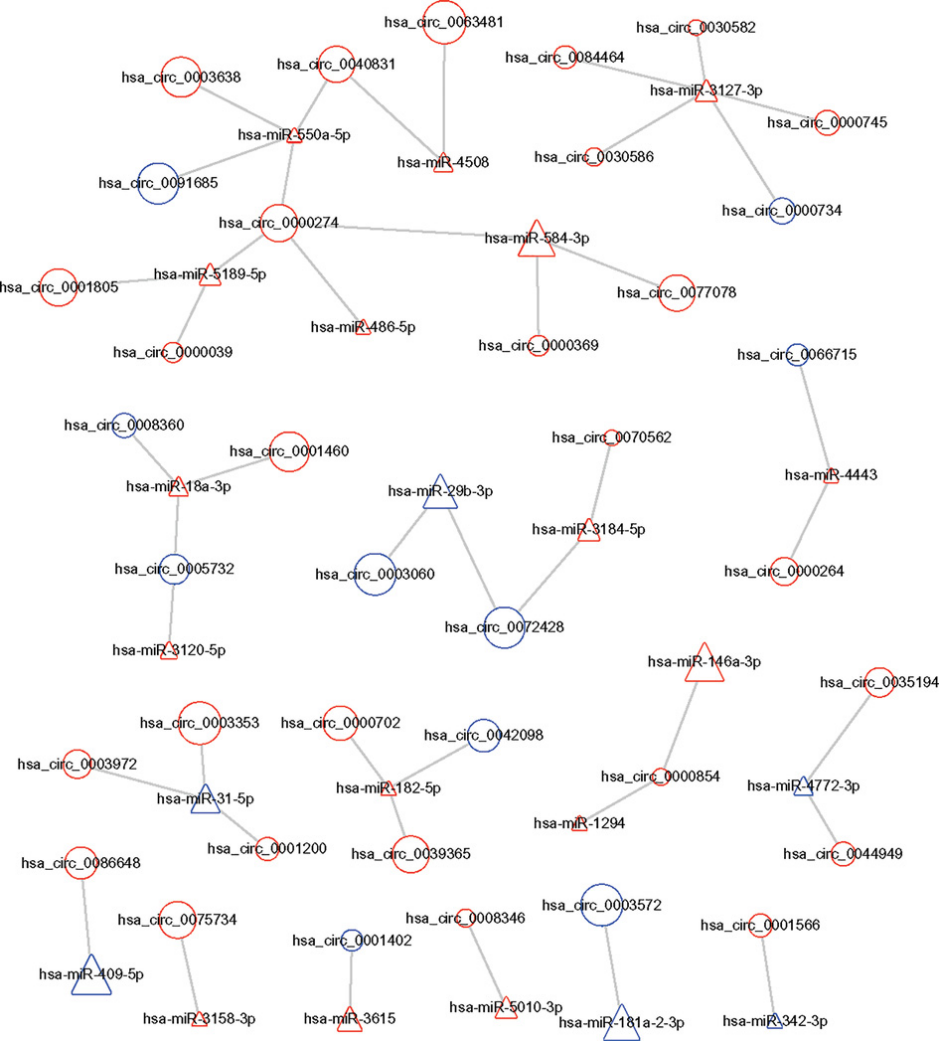
CircRNA–miRNA co-expression network Circles and triangles represent circRNAs and miRNAs, respectively. Red and blue represent upregulation and downregulation, respectively. Node size represents the *P* value, the bigger the node, the smaller the *P* value.

**Figure 4:**
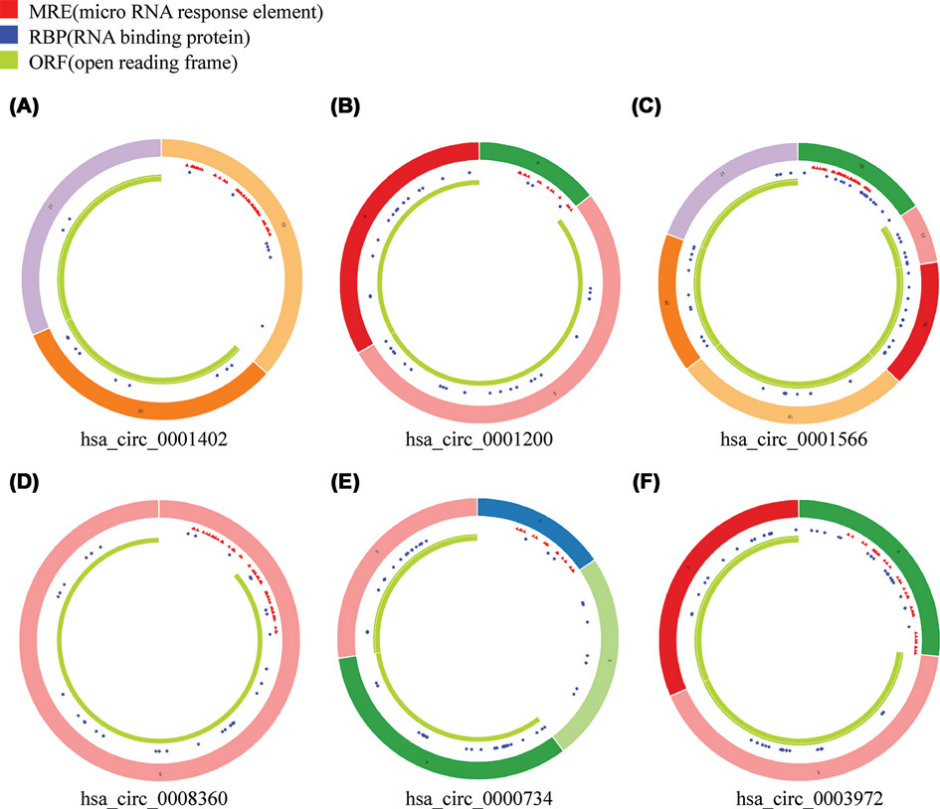
Structural patterns of the six circRNAs by the Cancer-Specific CircRNA (CSCD, http://gb.whu.edu.cn/CSCD/) (**A**) hsa circ 0001402, (**B**) hsa circ 0001200, (**C**) hsa circ 0001566, (**D**) hsa circ 0008360, (**E**) hsa circ 0000734, (**F**) hsa circ 0003972.

By using CircInteractome database (https://circinteractome.nia.nih.gov/), we predicted the binding site relationship between the differentially expressed miRNAs and differentially expressed circRNAs. To identify the possible modulating mechanisms of the circRNAs, a circRNA–miRNA co-expression network analysis was constructed by Cytoscape software (Figure 3). This procedure resulted in a complex circRNA target network that consisted of 228 matched circRNA–miRNA pairs for 165 differentially expressed circRNAs and 63 differentially expressed miRNAs. We selected six significantly changed circRNAs for further study, including three up-regulated hsa_-_circRNAs (0001200, 0001566, and 0003972) and three down-regulated hsa___circRNAs (0008360, 0000734 and 0001402). These genes are involved in apoptosis, autophagy, immunity, inflammation and oxidative stress by bioinformatics analysis. These process closely associated with RA. The basic characteristics of the six circRNAs are listed in Table 2. The basic structural patterns of these circRNAs are shown in Figure 4.

**Table 2:** Basic characteristics of the six differently expressed circRNAs

circRNAs	Position	*P*	Fold change	Regulation	Gene Symbol
hsa_circ_0001200	chr21:46275124-46281186	0.0146	2.26	Up-regulation	PTTG1IP
hsa_circ_0001566	chr5:179688683-179707608	0.0146	2.26	Up-regulation	MAPK9
hsa_circ_0003972	chr9:96238537-96261168	0.0239	2.10	Up-regulation	FAM120A
hsa_circ_0008360	chr22:41277773-41278181	0.0168	-2.16	Down-regulation	XPNPEP3
hsa_circ_0000734	chr17:1746096-1756483	0.0197	-1.80	Down-regulation	RPA1
hsa_circ_0001402	chr4:38091552-38104778	0.0116	-1.28	Down-regulation	TBC1D1

### Quantitative RT-qPCR validation of the differentially expressed circRNAs

**Figure 5:**
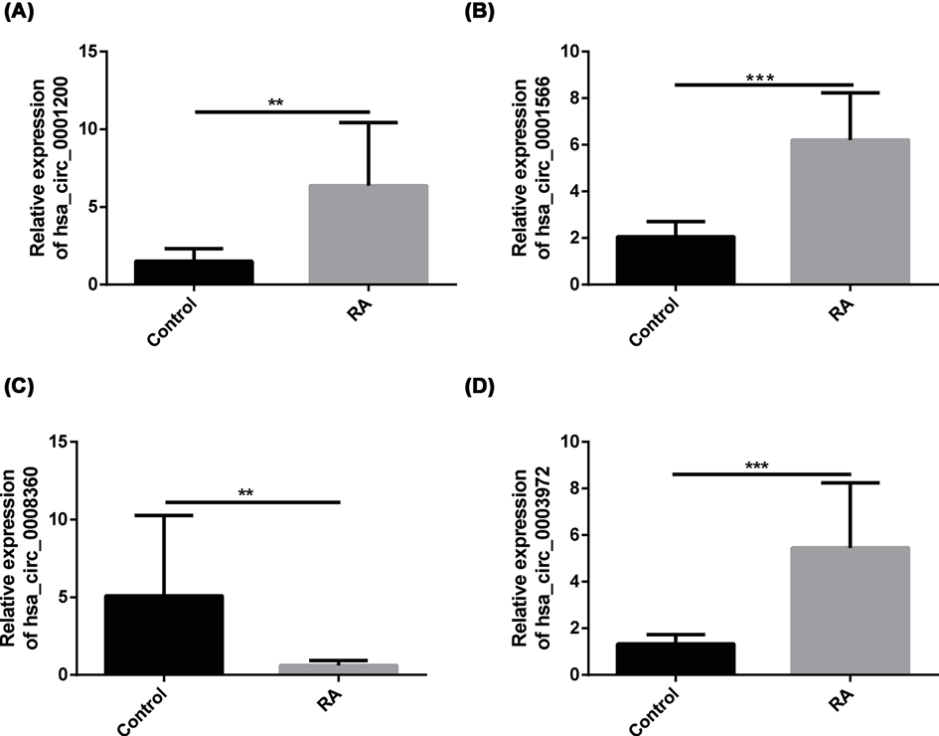
Quantitative RT-qPCR determined the relative expression levels of circRNAs in PBMCs from 10 RA patients and 10 healthy controls Hsa_circ_0001200 (*P*<0.01), hsa_circ_0001566 (*P*<0.01), hsa_circ_0003972 (*P*<0.01) exhibited the same increasing trend as the sequencing results (**A,B,D**). Hsa_circ_0008360 (*P*<0.01) exhibited the same decreasing trend as the sequencing results (**C**). A p-value < 0.05 was statistically significant (*P<0.05, **P<0.01, ***P<0.001).

To validate the sequencing expression data, we performed RT-qPCR on the six candidate circRNAs (Table 2) using an independent set of samples from 10 RA patients and 10 healthy people. The primers used are shown in Table 3. Consistent with the sequencing data, the average expression levels of hsa_circ_0001200, hsa_circ_0001566 and hsa_circ_0003972 in the PBMCs of patients with RA were significantly higher than those of the healthy people, hsa_circ_0008360 was significantly lower in patients with RA than in healthy people (Figure 5).

**Table 3:** Specific circRNAs primers used for quantitative RT-qPCR analysis

Gene name	Sequence
GAPDH	F: GGAGCGAGATCCCTCCAAAAT
	R: GGCTGTTGTCATACTTCTCATGG
hsa_circ_0001200	F: CGGACAGGAGTGAGGAGAAG
	R: TGGCAAGACGCTTGTAACTG
hsa_circ_0001566	F: CATGGAGCTGGATCATGAAA
	R: AGGTTGAGTCTGCCACTTGC
hsa_circ_0003972	F: AGGAAATCACATTCTGCCTGA
	R: CAACGGCTTTGATCACTACG
hsa_circ_0008360	F: TCGAGAACTTTGGGATGGTC
	R: TTTGTGTCTGCGAAGTGCAT
hsa_circ_0000734	F: CAGCTGAAGCAGTTGGAGTG
	R: GATAACGCGGCGGACTATT
hsa_circ_0001402	F: AAACAGCAGCCAAAGGATGT
	R: GCCCATCTTCACAAACTGGT

### Spearman correlation test of clinical variables and confirmed circRNAs in PBMCs from RA patients

**Figure 6:**
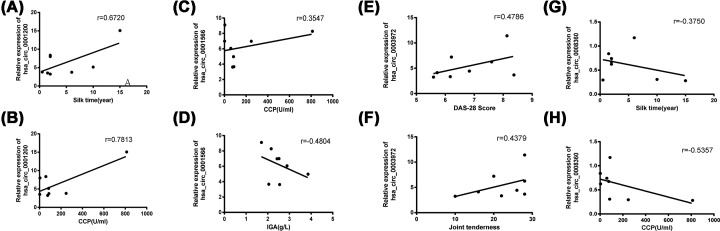
Correlation between circRNAs and clinical disease activities There was close positive correlation of the hsa_circ_0001200 levels with silk time and CCP levels in RA (**A** and **B**). There was a close positive correlation of the hsa_circ_0001566 level with the CCP (**C**) and a negative correlation with IGA levels in RA (**D**). There was a close positive correlation of the hsa_circ_0003972 levels with DAS28 and joint tenderness in RA (**E** and **F**). There was a close negative correlation between the hsa_circ_0008360 level with the silk time and CCP (**G** and **H**).

To determine whether the significantly and differentially expressed circRNAs in the RA patients were relevant biomarkers for the disease activity of RA, we performed Spearman correlation tests to assess the correlation between RA clinical features (ESR, hs-CRP, RF, CCP, IGA, IGG, IGM, C3, C4, DAS28, VAS, SAS, SDS, etc.) and circRNA levels (hsa_circ_0001200, hsa_circ_0001566, hsa_circ_0003972 and hsa_circ_0008360). We found that the hsa_circ_0001200 levels were positively associated with silk time and CCP levels in RA (Figure 6A,B), the hsa_circ_0001566 level was positively associated with CCP (Figure 6C) and negatively associated with IGA (Figure 6D), the hsa_circ_0003972 levels were positively associated with DAS28 and joint tenderness in RA (Figure 6E,F), and the hsa_circ_0008360 level was negatively associated with the silk time and CCP (Figure 6E–H).

## Discussion

RA is a systemic autoimmune disease with unknown etiology. In previous decades, great efforts have been made to explore the molecular mechanisms of RA [13,14]; however, the majority of previous studies have concentrated on protein-coding genes. Rapidly accumulating evidence has now suggested that although noncoding RNAs do not directly encode proteins, they play a regulatory role in the transcription and translation of protein-coding genes [15]. There are many previous studies showing that lncRNAs participate in the pathogenesis of RA and can be regarded as biomarkers for the diagnosis of RA [16,17]. Recently, there is increasing evidence proving that circRNAs are widely involved in various physiological and pathological processes, including the occurrence and development of many diseases, including lung cancer [18], liver cancer [19], gynecological diseases [20] and more.

Little research about the role of circRNAs in RA has been conducted. Several recent studies have shown that hsa_circ_0044235, hsa_circ_RNA_104871 and hsa_circ_RNA_003524 in PBMCs participate in the pathogenesis of RA and can be used as potential biomarkers of RA by microarray analysis [21,22]. In our study, we successfully explored the circRNAs and miRNAs expression profile in PBMCs of RA patients and healthy people using sequencing technology, then evaluated circRNAs expression and circRNA–miRNA co-expression in RA patients. Based on the sequencing data, we identified 165 circRNAs and 63 miRNAs that were differentially expressed between the two groups, suggesting that a large number of circRNAs may be related to the RA disease process. Our results may enrich the study of the pathogenesis of RA and provide a theoretical basis for the in-depth exploration of the function of circRNAs in RA.

To study the biological functions of these circRNAs, we used GO analysis and KEGG pathway analysis to explore their biological functions. According to the GO analysis, the main biological processes involving the differentially expressed circRNAs included cellular protein modification processes and cellular metabolism processes. Pathway analysis showed that the genes associated with the differentially expressed circRNAs mainly involved the ErbB, TGF-β, TNF and FoxO signaling pathways.

Some previous research found that reduced FoxO1 expression is required to promote broblast-like synoviocytes survival in RA [23]. Moreover, another study showed that inhibition of miR21 through the TGFβ1/Smad4/7 signaling pathway can down-regulate the expression of MMP1, MMP3 and MMP13, and finally suppress the invasiveness of fibroblast-like synoviocytes in RA [24]. Surprisingly, we have also found some enriched pathways for which the roles in the development of RA have not previously been researched, such as the sphingolipid signaling pathway and Epstein–Barr virus infection, which need to be studied more deeply.

To the best of our knowledge, circRNAs play an important biological role via a variety of mechanisms, including chromatin modification, splicing and more. Additionally, an increasing amount of evidence has shown that circRNAs may play a critical regulatory function in RA through a co-expression network. Thus, we constructed a circRNA–miRNA co-expression network to identify the circRNAs associated with RA.

To verify the dependability of the sequencing data, we chose three up-regulated (hsa-circRNA0001200, hsa-circRNA0001566 and hsa-circRNA0003972) and three down-regulated (hsa-circRNA0008360, hsa-circRNA0000734, and hsa_circ_0001402) circRNAs for validation by RT-qPCR in 10 RA patients compared with 10 healthy people. These genes are enriched in the pathways of apoptosis, autophagy, immunity, inflammation and oxidative stress, which are closely related to the occurrence of RA. As a consequence, we found that hsa_circ_0001200 (*P* < 0.01), hsa_circ_0001566 (*P* < 0.01) and hsa_circ_0003972 (*P* < 0.01) were indeed up-regulated, whereas hsa_circ_0008360 (*P* < 0.01) was indeed down-regulated in RA compared with healthy subjects. These results were consistent with the RNA-seq. A recent study showed that hsa_circ_0044235 in PBMCs may be a potential biomarker of RA patients [21]. Another study suggests that increased expression of circRNAs circRNA_104871, circRNA_003524, circRNA_101873 and circRNA_103047 in PBMC from RA patients may serve as potential biomarkers for RA patient diagnosis [22]. Furthermore, we observed correlations between the expression of hsa_circ_0001200, hsa_circ_0001566, hsa_circ_0003972, and hsa_circ_0008360 with biochemical measurements. The results showed that the hsa_circ_0001200 levels were positively associated with silk time and CCP levels in RA, the hsa_circ_0001566 level was positively associated with CCP and negatively associated with IGA, the hsa_circ_0003972 levels were positively associated with DAS28 and joint tenderness in RA, and the hsa_circ_0008360 level was negatively associated with the silk time and CCP. These results reveal that hsa_circ_0001200, hsa_circ_0001566, hsa_circ_0003972 and hsa_circ_0008360 may play critical roles in the pathogenesis mechanism of RA.

As a chronic disease, RA can have significant effects on both physical and psychosocial health, and it adversely affects the quality of life of the patients. Previous studies have investigated the effects of RA itself on patients’ quality of life, depression, and anxiety [25,26]. Future studies may confirm the molecular regulatory mechanisms of these candidate circRNAs.

We recognize some limitations in the present study. First, the sample size was small and a larger sample should be collected from different regions and different races. Second, we just identified circRNAs potentially associated with RA, and the molecular mechanisms of these potential regulators in RA need to be studied. Third, to determine whether hsa_circ_0001200, hsa_circ_0001566, hsa_circ_0003972 and hsa_circ_0008360 can be diagnostic biomarkers, we need to evaluate their ability to effectively distinguish RA from other rheumatic diseases, such as ankylosing spondylitis (AS), systemic lupus erythematosus (SLE) and osteoarthritis (OA). These other rheumatic diseases should be included in future studies to reinforce the argument that hsa_circ_0001200, hsa_circ_0001566, hsa_circ_0003972 and hsa_circ_0008360 can be used as diagnostic biomarkers for RA.

## Supplementary Material

Supplementary Tables S1-S2

## Abbreviations

AS: ankylosing spondylitis
circRNA: circular RNA
miRNA: microRNA
OA: osteoarthritis
PBMC: peripheral blood mononuclear cell
RA: rheumatoid arthritis
SLE: systemic lupus erythematosus
