# Supplementary material for: RNA-seq reveals the circular RNA and miRNA expression profile of peripheral blood mononuclear cells in patients with rheumatoid arthritis
Source: Biosci Rep. 2020 Apr 3;40(4):BSR20193160. doi: 10.1042/BSR20193160 (PMC7133114; doi:10.1042/BSR20193160)
Supplement: Supplementary Tables S1-S2 [file BSR-2019-3160_supp.pdf]

Table S1 Differentially expressed circRNAs in

cirRNA_ID	log2FoldChange	Pvalue	circRNA_t	transcript_id
10:124038515|124C	2.10	0.023082491	exon	ENST00000346248,EN
7:102877467|10288	-1.47	0.03473953	exon	ENST00000448002,EN
5:41794003|418074	2.31	0.010837877	exon	ENST00000509987,EN
4:38089932|381031	-1.28	0.011579354	exon	ENST00000508802,EN
1:172555869|17255	2.93	0.002726106	exon	ENST00000610051,EN
1:155438327|15545	2.11	0.012940879	exon	ENST00000392403,EN
20:34470048|34481	-1.84	0.015237536	exon	ENST00000262650,EN
10:32543300|32584	-1.92	0.042841678	exon	ENST00000639629
11:128758115|1287	2.32	0.010184065	exon	ENST00000281428,EN
15:68141946|68164	2.10	0.022680688	exon	ENST00000545237,EN
10:84417771|84425	-1.31	0.025606836	exon	ENST00000224756,EN
4:105233897|10523	2.50	0.001558809	exon	ENST00000380013,EN
17:20204333|20205	1.76	0.017587862	exon	ENST00000395522,EN
6:75621532|756348	1.94	0.037551415	exon	ENST00000493959,EN
9:33944365|339561	1.98	0.024253069	exon	ENST00000379238,EN
16:30496097|30496	-1.84	0.011339098	exon	ENST00000358164,EN
9:83768818|837886	2.22	0.014942916	exon	ENST00000376371
6:13632370|136447	1.18	0.03860946	exon	ENST00000011619
17:47370437|47379	-2.88	0.002246282	exon	ENST00000331493,EN
5:50750151|507972	1.94	0.038334603	exon	ENST00000503561,EN
13:28197185|2822C	-1.67	0.03135271	exon	ENST00000503791,EN
17:29467145|29482	1.61	0.048390904	exon	ENST00000261716,EN
9:121161856|12116	-1.62	0.043254675	exon	ENST00000373845,EN
2:203402576|20342	1.63	0.046394831	exon	ENST00000417864,EN
22:41125864|4114C	2.79	0.025441411	exon	ENST00000263253
2:156549608|15655	-1.95	0.026263393	exon	ENST00000310454,EN
8:123077111|12308	-1.40	0.042721232	exon	ENST00000518099,EN
11:32927157|32935	2.36	0.036577939	exon	ENST00000399302,EN
10:89342414|89342	-1.97	0.037081415	intron	ENST00000463623,EN
4:122927634|12297	1.94	0.038768562	exon	ENST00000422835,EN
10:1124218|112502	-1.90	0.037672333	exon	ENST00000263150,EN
20:41533050|41551	1.86	0.039097508	exon	ENST00000373233
13:110622216|1106	1.87	0.03824319	exon	ENST00000470164,EN
5:180261684|18028	2.26	0.014573786	exon	ENST00000455781,EN
22:46626751|46637	-2.25	0.02340708	exon	ENST00000406902
18:62539681|6255C	1.55	0.003787232	exon	ENST00000586834,EN
2:119926598|11993	2.79	0.003527997	exon	ENST00000263708,EN
1:243613671|24369	-2.25	0.011224785	exon	ENST00000366540,EN
2:33134793|331888	2.74	0.004619893	exon	ENST00000404816,EN
14:50640750|50645	1.94	0.038334603	exon	ENST00000324679,EN
8:67103037|67132C	1.86	0.039097508	exon	ENST00000519668,EN
13:95161189|95188	2.34	0.006320114	exon	ENST00000376887,EN
X:150727215|1507	-1.54	0.045645239	exon	ENST00000485376,EN
17:28163543|28172	1.19	0.043831574	exon	ENST00000407008,EN
4:84739010|847404	1.73	0.041127712	exon	ENST00000295888

10:15833630|15843	1.94	0.037551415	exon	ENST00000277632,EN
2:171028339|17106	1.61	0.048390904	exon	ENST00000360843,EN
2:8943187|8974435	2.10	0.041276773	exon	ENST00000462696
6:17665239|176757	1.94	0.038334603	exon	ENST00000537253,EN
11:128480191|1284	-1.52	0.049155195	exon	ENST00000319397,EN
2:40428473|404303	1.67	0.011832172	exon	ENST00000402441,EN
2:33110582|331349	1.87	0.03824319	exon	ENST00000404816
16:88627622|88628	1.79	0.037254331	exon	ENST00000301011,EN
11:3731391|375340	1.86	0.039097508	exon	ENST00000529379,EN
2:230442937|23045	1.56	0.030093679	exon	ENST00000409112,EN
14:73147795|73148	2.10	0.023712476	exon	ENST00000324501,EN
8:55966715|559697	-2.12	0.012461268	exon	ENST00000420292,EN
14:103778745|1037	-1.75	0.026610126	exon	ENST00000202556,EN
20:41092472|41101	2.19	0.003825742	exon	ENST00000361337
17:60265412|60294	2.08	0.016037108	exon	ENST00000300896,EN
5:95755396|957636	1.22	0.03225468	exon	ENST00000379982
21:29321221|29329	2.50	0.000293728	exon	ENST00000286800,EN
3:170136419|17014	1.82	0.049842871	exon	ENST00000479467,EN
18:21023620|21042	-1.54	0.045645239	exon	ENST00000399799,EN
5:61472681|614730	2.08	0.042263199	exon	ENST00000252744
2:61498673|615339	1.86	0.039097508	exon	ENST00000406957,EN
1:168038371|16806	1.63	0.04691439	exon	ENST00000312263,EN
12:12519862|12521	-1.86	0.029487422	exon	ENST00000298573,EN
8:30101898|301044	-2.36	0.015726641	exon	ENST00000442880,EN
1:8655973|8656441	2.06	0.031931196	exon	ENST00000400908,EN
15:65752379|65761	2.12	0.04156796	exon	ENST00000564674,EN
10:31908172|31910	-1.62	0.041695408	exon	ENST00000344936,EN
15:65715478|65761	2.48	0.007000752	exon	ENST00000564674,EN
17:1842803|185318	-1.80	0.019744373	exon	ENST00000570451,EN
9:19360244|193619	-1.67	0.03135271	exon	ENST00000361024,EN
4:139125164|13913	2.18	0.018105313	exon	ENST00000394235,EN
9:33318731|333191	1.77	0.030134953	exon	ENST00000379540,EN
22:42411407|42411	2.10	0.023943271	exon	ENST00000329021,EN
4:105446383|10545	-1.90	0.007840422	exon	ENST00000513649,EN
5:39201826|392029	-1.54	0.045645239	exon	ENST00000351578,EN
11:85996826|86003	1.11	0.035629228	exon	ENST00000532317,EN
13:95115922|95188	2.97	0.001559041	exon	ENST00000376887,EN
17:31940286|31966	2.10	0.02351929	exon	ENST00000322652,EN
12:827042|830160	1.98	0.024253069	exon	ENST00000537687,EN
1:39853970|398574	-2.16	0.01676277	exon	ENST00000492612,EN
22:40881770|40882	-2.16	0.01676277	exon	ENST00000428799,EN
4:177353308|17736	1.21	0.043357576	exon	ENST00000264596
2:43578513|435909	-1.52	0.047920544	exon	ENST00000405006,EN
16:53255600|53268	1.94	0.038334603	exon	ENST00000615216,EN
20:35729312|35732	1.75	0.041814061	exon	ENST00000639702,EN
1:172551512|17255	1.68	0.010975143	exon	ENST00000610051,EN
17:12095575|12113	-1.61	0.03113567	exon	ENST00000538465,EN
1:236859833|23686	2.10	0.023712476	exon	ENST00000366576,EN
5:180549931|18055	2.26	0.015266036	exon	ENST00000261951,EN
16:70532479|70544	2.08	0.016037108	exon	ENST00000302516

1:93894614|938978	2.44	0.025625536	exon	ENST00000615724,EN
8:38114192|381211	2.16	0.011138676	exon	ENST00000517496,EN
5:71502599|715124	1.51	0.049240057	exon	ENST00000508917,EN
7:152783187|15280	1.61	0.049995497	exon	ENST00000377776,EN
3:184924862|18498	1.94	0.038334603	exon	ENST00000436792,EN
3:105670233|10569	-2.12	0.012461268	exon	ENST00000264122,EN
1:151658235|15166	1.61	0.049995497	exon	ENST00000368838,EN
10:84417771|84438	-2.01	0.044803628	exon	ENST00000224756,EN
4:78826037|788788	2.12	0.032146549	exon	ENST00000389010,EN
4:77742141|777763	-1.95	0.026263393	exon	ENST00000504123,EN
5:80767933|807928	2.26	0.015266036	exon	ENST00000265081
1:176081152|17613	-1.71	0.046892869	exon	ENST00000367669,EN
13:114041000|1140	-1.71	0.046892869	exon	ENST00000334062
11:14294471|14295	-2.32	0.011014659	exon	ENST00000529237,EN
6:75659262|756789	2.26	0.014573786	exon	ENST00000370010,EN
15:63529014|63574	2.22	0.014702415	exon	ENST00000558157
2:187484124|18750	1.61	0.048390904	exon	ENST00000392365,EN
6:13579451|135842	-2.20	0.030725244	exon	ENST00000397350,EN
4:145159503|14517	1.75	0.041434525	exon	ENST00000455611,EN
18:32042179|32045	-1.71	0.046892869	exon	ENST00000580863,EN
4:143415477|14344	1.92	0.040425931	exon	ENST00000262995,EN
3:151116338|15112	2.31	0.010837877	exon	ENST00000422248,EN
21:44855210|44861	2.26	0.014573786	exon	ENST00000397886,EN
2:226864604|22690	2.10	0.02351929	exon	ENST00000341329,EN
14:101901719|1019	2.06	0.045290633	exon	ENST00000445439,EN
10:103347238|1033	-1.90	0.037672333	exon	ENST00000490296,EN
17:30481143|30492	-1.52	0.049155195	exon	ENST00000451249,EN
18:2585113|261086	1.72	0.040399963	exon	ENST00000261597
10:96907265|96952	1.76	0.031050571	exon	ENST00000421806,EN
16:30483827|30484	-1.10	0.028325032	exon	ENST00000358164,EN
5:168488602|16849	2.49	0.042998131	exon	ENST00000520013,EN
22:41125864|41146	1.51	0.049240057	exon	ENST00000263253
1:180041138|18004	-1.69	0.029368926	exon	ENST00000367607
10:11997672|12014	1.71	0.019830209	exon	ENST00000357604,EN
14:58215205|58223	-1.52	0.049155195	exon	ENST00000556748,EN
17:47370437|47377	-2.04	0.016775564	exon	ENST00000331493,EN
15:41356039|41377	2.39	0.005886739	exon	ENST00000450592,EN
9:130079630|13009	-1.54	0.045645239	exon	ENST00000372410,EN
19:54909245|54910	-1.81	0.026900725	exon	ENST00000598576,EN
15:49625113|49634	1.98	0.025575102	exon	ENST00000558653,EN
15:65729072|65756	2.70	0.017691075	exon	ENST00000564674,EN
11:86012281|86031	1.86	0.039097508	exon	ENST00000532317,EN
17:55401469|55403	1.96	0.031985716	exon	ENST00000571578,EN
7:48410520|484125	1.98	0.032492642	exon	ENST00000435803,EN
5:141580744|14158	-1.49	0.043272334	exon	ENST00000523100,EN
5:50402286|504113	-1.54	0.045645239	exon	ENST00000303221,EN
16:47497399|47515	1.30	0.002763599	exon	ENST00000323584,EN
12:32598497|32611	1.51	0.00712282	exon	ENST00000551984,EN
7:152263016|15226	1.86	0.040746277	exon	ENST00000558084,EN
19:18537601|18538	1.40	0.027553571	exon	ENST00000608443,EN

7:112286873|11228	-1.95	0.026263393	exon	ENST00000450657,EN
X:19965044|19970	1.63	0.046394831	exon	ENST00000379687,EN
1:28742403|287439	2.46	0.009548171	exon	ENST00000541996,EN
9:4823548|4833228	2.16	0.011138676	exon	ENST00000381750,EN
12:32607957|32611	1.91	0.02267251	exon	ENST00000551984,EN
18:45988702|45992	-2.05	0.024008024	exon	ENST00000409746
16:4466153|446946	-2.12	0.033286002	exon	ENST00000404295,EN
X:154789954|1547	1.76	0.031050571	exon	ENST00000369534,EN
2:37974040|380042	1.76	0.031050571	exon	ENST00000406384,EN
16:53254438|53274	2.04	0.034291481	exon	ENST00000615216,EN
12:9584106|960780	1.87	0.03824319	exon	ENST00000641304,EN
9:83677727|836861	2.16	0.011138676	exon	ENST00000257468,EN
18:70121556|70128	-1.47	0.03473953	exon	ENST00000581161,EN
8:47953620|479574	2.34	0.01434032	exon	ENST00000314191,EN
9:93476256|934988	2.10	0.023943271	exon	ENST00000375389,EN
2:230357805|23036	-2.05	0.024008024	exon	ENST00000415673,EN
16:29355787|29365	-1.54	0.045645239	exon	ENST00000507381,EN
2:120278618|12028	1.61	0.048390904	exon	ENST00000420510,EN
3:67495798|674982	-1.71	0.046892869	exon	ENST00000493112,EN
8:140818277|14086	1.73	0.041127712	exon	ENST00000340930,EN

ı RA by RNA-seq analysis

gene_id	Symbol	Description	cirbase_id
ENSG00000182022	CHST15	carbohydrate sulfotrans	hsa_circ_0000264
ENSG00000161040	FBXL13	F-box and leucine rich ı	---
ENSG00000083720	OXCT1	3-oxoacid CoA-transfer	hsa_circ_0004873
ENSG00000065882	TBC1D1	TBC1 domain family m	hsa_circ_0001402
ENSG00000094975	SUCO	SUN domain containing	hsa_circ_0015262
ENSG00000116539	ASH1L	ASH1 like histone lysin	hsa_circ_0003247
ENSG00000078747	ITCH	itchy E3 ubiquitin prote	hsa_circ_0005868
ENSG00000216937	CCDC7	coiled-coil domain cont	hsa_circ_0000233
ENSG00000151702	FLI1	Fli-1 proto-oncogene, E	hsa_circ_0000369
ENSG00000033800	PIAS1	protein inhibitor of acti	hsa_circ_0036044
ENSG00000107771	CCSER2	coiled-coil serine rich p	hsa_circ_0006956
ENSG00000168769	TET2	tet methylcytosine diox	hsa_circ_0070562
ENSG00000128487	SPECC1	sperm antigen with calp	hsa_circ_0000745
ENSG00000112701	SENP6	SUMO1/sentrin specifi	hsa_circ_0077078
ENSG00000137073	UBAP2	ubiquitin associated prc	hsa_circ_0001846
ENSG00000005844	ITGAL	integrin subunit alpha L	---
ENSG00000165113	GKAP1	G kinase anchoring pro	---
ENSG00000010017	RANBP9	RAN binding protein 9	hsa_circ_0001577
ENSG00000178852	EFCAB13	EF-hand calcium bindir	hsa_circ_0044242
ENSG00000151883	PARP8	poly(ADP-ribose) polyı	hsa_circ_0072431
ENSG00000152520	PAN3	PAN3 poly(A) specific	hsa_circ_0006597
ENSG00000160551	TAOK1	TAO kinase 1	---
ENSG00000119397	CNTRL	centriolin	hsa_circ_0088333
ENSG00000138443	ABI2	abl interactor 2	hsa_circ_0007951
ENSG00000100393	EP300	E1A binding protein p3	---
ENSG00000115159	GPD2	glycerol-3-phosphate de	hsa_circ_0005732
ENSG00000156787	TBC1D31	TBC1 domain family m	hsa_circ_0085438
ENSG00000060749	QSER1	glutamine and serine ric	hsa_circ_0021570
ENSG00000107798	LIPA	lipase A, lysosomal aci	---
ENSG00000145375	SPATA5	spermatogenesis associ	---
ENSG00000047056	WDR37	WD repeat domain 37	hsa_circ_0017469
ENSG00000124177	CHD6	chromodomain helicase	hsa_circ_0001159
ENSG00000213995	NAXD	NAD(P)HX dehydratas	hsa_circ_0030883
ENSG00000050748	MAPK9	mitogen-activated prote	hsa_circ_0001566
ENSG00000075240	GRAMD4	GRAM domain contain	hsa_circ_0001250
ENSG00000141664	ZCCHC2	zinc finger CCHC-type	hsa_circ_0000854
ENSG00000088179	PTPN4	protein tyrosine phosph	hsa_circ_0056247
ENSG00000117020	AKT3	AKT serine/threonine k	hsa_circ_0017251
ENSG00000049323	LTBP1	latent transforming gro	---
ENSG00000151748	SAV1	salvador family WW dc	hsa_circ_0007101
ENSG00000104218	CSPP1	centrosome and spindle	hsa_circ_0001805
ENSG00000125257	ABCC4	ATP binding cassette sı	hsa_circ_0030586
ENSG00000063601	MTMR1	myotubularin related pr	hsa_circ_0091685
ENSG00000087095	NLK	nemo like kinase	hsa_circ_0003638
ENSG00000163625	WDFY3	WD repeat and FYVE c	hsa_circ_0070323

ENSG00000148481	MINDY3	MINDY lysine 48 deub	hsa_circ_0005825
ENSG00000198586	TLK1	tousled like kinase 1	---
ENSG00000143797	MBOAT2	membrane bound O-acy	---
ENSG00000124789	NUP153	nucleoporin 153	hsa_circ_0075734
ENSG00000134954	ETS1	ETS proto-oncogene 1,	hsa_circ_0002083
ENSG00000183023	SLC8A1	solute carrier family 8 r	hsa_circ_0000994
ENSG00000049323	LTBP1	latent transforming grov	---
ENSG00000158545	ZC3H18	zinc finger CCCH-type	hsa_circ_0040831
ENSG00000110713	NUP98	nucleoporin 98	hsa_circ_0000274
ENSG00000067066	SP100	SP100 nuclear antigen	hsa_circ_0003922
ENSG00000080815	PSEN1	presenilin 1	hsa_circ_0003848
ENSG00000254087	LYN	LYN proto-oncogene, S	hsa_circ_0084552
ENSG00000088808	PPP1R13B	protein phosphatase 1 r	hsa_circ_0005791
ENSG00000198900	TOP1	topoisomerase (DNA) I	---
ENSG00000170832	USP32	ubiquitin specific pepti	hsa_circ_0044949
ENSG00000164292	RHOBTB3	Rho related BTB doma	hsa_circ_0007444
ENSG00000156273	BACH1	BTB domain and CNC	hsa_circ_0001181
ENSG00000173889	PHC3	polyhomeotic homolog	hsa_circ_0001965
ENSG00000067900	ROCK1	Rho associated coiled-c	---
ENSG00000130449	ZSWIM6	zinc finger SWIM-type	hsa_circ_0005399
ENSG00000082898	XPO1	exportin 1	hsa_circ_0054894
ENSG00000143164	DCAF6	DDB1 and CUL4 assoc	hsa_circ_0015132
ENSG00000111266	DUSP16	dual specificity phospha	hsa_circ_0003855
ENSG00000104660	LEPROTL1	leptin receptor overlapp	hsa_circ_0007353
ENSG00000142599	RERE	arginine-glutamic acid (	---
ENSG00000174485	DENND4A	DENN domain containi	hsa_circ_0035957
ENSG00000165322	ARHGAP12	Rho GTPase activating	hsa_circ_0000231
ENSG00000174485	DENND4A	DENN domain containi	---
ENSG00000132383	RPA1	replication protein A1	hsa_circ_0000734
ENSG00000137145	DENND4C	DENN domain containi	hsa_circ_0007110
ENSG00000109381	ELF2	E74 like ETS transcript	hsa_circ_0001441
ENSG00000086102	NFX1	nuclear transcription fa	hsa_circ_0086648
ENSG00000235568	NFAM1	NFAT activating protei	hsa_circ_0001240
ENSG00000138777	PPA2	pyrophosphatase (inorg	hsa_circ_0007477
ENSG00000082074	FYB1	FYN binding protein 1	hsa_circ_0072340
ENSG00000073921	PICALM	phosphatidylinositol bii	hsa_circ_0023919
ENSG00000125257	ABCC4	ATP binding cassette su	hsa_circ_0030582
ENSG00000178691	SUZ12	SUZ12 polycomb repre	hsa_circ_0042963
ENSG00000060237	WNK1	WNK lysine deficient p	hsa_circ_0024960
ENSG00000043514	TRIT1	tRNA isopentenyltransf	hsa_circ_0000057
ENSG00000196236	XPNPEP3	X-prolyl aminopeptidas	hsa_circ_0008360
ENSG00000109674	NEIL3	nei like DNA glycosyla	hsa_circ_0001460
ENSG00000115970	THADA	THADA, armadillo rep	hsa_circ_0054322
ENSG00000177200	CHD9	chromodomain helicase	hsa_circ_0039365
ENSG00000131051	RBM39	RNA binding motif pro	hsa_circ_0008817
ENSG00000094975	SUCO	SUN domain containing	hsa_circ_0000160
ENSG00000065559	MAP2K4	mitogen-activated prote	hsa_circ_0042098
ENSG00000116984	MTR	5-methyltetrahydrofolat	hsa_circ_0003929
ENSG00000113300	CNOT6	CCR4-NOT transcriptic	hsa_circ_0008836
ENSG00000189091	SF3B3	splicing factor 3b subur	hsa_circ_0005330

ENSG00000023909	GCLM	glutamate-cysteine ligas	hsa_circ_0013222
ENSG00000129691	ASH2L	ASH2 like histone lysir	hsa_circ_0006302
ENSG00000145734	BDP1	B double prime 1, subu	---
ENSG00000133627	ACTR3B	ARP3 actin related prot	hsa_circ_0006890
ENSG00000156931	VPS8	VPS8, CORVET compl	---
ENSG00000114423	CBLB	Cbl proto-oncogene B	hsa_circ_0066715
ENSG00000143376	SNX27	sorting nexin family me	hsa_circ_0002394
ENSG00000107771	CCSER2	coiled-coil serine rich p	hsa_circ_0003018
ENSG00000138756	BMP2K	BMP2 inducible kinase	---
ENSG00000138767	CNOT6L	CCR4-NOT transcriptic	---
ENSG00000113318	MSH3	mutS homolog 3	hsa_circ_0073177
ENSG00000143207	RFWD2	ring finger and WD rep	hsa_circ_0003572
ENSG00000185989	RASA3	RAS p21 protein activa	hsa_circ_0031071
ENSG00000133818	RRAS2	related RAS viral (r-ras	hsa_circ_0004429
ENSG00000112701	SENP6	SUMO1/sentrin specifi	---
ENSG00000140455	USP3	ubiquitin specific pepti	---
ENSG00000003436	TFPI	tissue factor pathway in	hsa_circ_0003353
ENSG00000124523	SIRT5	sirtuin 5	hsa_circ_0007218
ENSG00000164164	OTUD4	OTU deubiquitinase 4	---
ENSG00000101695,EN	RNF125,AC01	ring finger protein 125,	---
ENSG00000109458	GAB1	GRB2 associated bindi	---
ENSG00000144893	MED12L	mediator complex subu	---
ENSG00000183255	PTTG1IP	PTTG1 interacting prot	hsa_circ_0001200
ENSG00000144468	RHBDD1	rhomboid domain conta	hsa_circ_0058494
ENSG00000078304	PPP2R5C	protein phosphatase 2 r	hsa_circ_0003831
ENSG00000156374	PCGF6	polycomb group ring fi	hsa_circ_0005052
ENSG00000108587	GOSR1	golgi SNAP receptor cc	hsa_circ_0042839
ENSG00000080986	NDC80	NDC80, kinetochore co	---
ENSG00000196233	LCOR	ligand dependent nucle	hsa_circ_0019316
ENSG00000005844	ITGAL	integrin subunit alpha L	hsa_circ_0000690
ENSG00000113643	RARS	arginyl-tRNA synthetas	hsa_circ_0001551
ENSG00000100393	EP300	E1A binding protein p3	hsa_circ_0063481
ENSG00000135837	CEP350	centrosomal protein 35(	hsa_circ_0006924
ENSG00000151461	UPF2	UPF2, regulator of nons	hsa_circ_0000213
ENSG00000131966	ACTR10	actin related protein 10	---
ENSG00000178852	EFCAB13	EF-hand calcium bindir	---
ENSG00000137804	NUSAP1	nucleolar and spindle as	hsa_circ_0008346
ENSG00000148358	GPR107	G protein-coupled recep	---
ENSG00000189430	NCR1	natural cytotoxicity trig	hsa_circ_0052166
ENSG00000104047	DTWD1	DTW domain containin	hsa_circ_0035194
ENSG00000174485	DENND4A	DENN domain containi	hsa_circ_0035946
ENSG00000073921	PICALM	phosphatidylinositol bir	hsa_circ_0023940
ENSG00000108960	MMD	monocyte to macrophag	hsa_circ_0002015
ENSG00000179869	ABCA13	ATP binding cassette su	---
ENSG00000131504	DIAPH1	diaphanous related forn	hsa_circ_0074323
ENSG00000170571	EMB	embigin	hsa_circ_0072428
ENSG00000102893	PHKB	phosphorylase kinase re	hsa_circ_0000698
ENSG00000139132	FGD4	FYVE, RhoGEF and PH	hsa_circ_0025843
ENSG00000055609	KMT2C	lysine methyltransferas	hsa_circ_0002932
ENSG00000105701	FKBP8	FK506 binding protein	hsa_circ_0000914

ENSG00000198839	ZNF277	zinc finger protein 277	hsa_circ_0001739
ENSG00000173681	BCLAF3	BCLAF1 and THRAP3	---
ENSG00000198492	YTHDF2	YTH N6-methyladenos	hsa_circ_0000039
ENSG00000120158	RCL1	RNA terminal phosphat	hsa_circ_0006134
ENSG00000139132	FGD4	FYVE, RhoGEF and PI	hsa_circ_0000390
ENSG00000152229	PSTPIP2	proline-serine-threonine	---
ENSG00000153406	NMRAL1	NmrA like redox sensor	hsa_circ_0007788
ENSG00000130830	MPP1	membrane palmitoylate	hsa_circ_0001952
ENSG00000115841	RMDN2	regulator of microtubul	hsa_circ_0054130
ENSG00000177200	CHD9	chromodomain helicase	hsa_circ_0000702
ENSG00000284634	AC092821.3	---	---
ENSG00000135018	UBQLN1	ubiquilin 1	hsa_circ_0003715
ENSG00000176225	RTTN	rotatin	---
ENSG00000253729	PRKDC	protein kinase, DNA-ac	hsa_circ_0084464
ENSG00000048828	FAM120A	family with sequence si	hsa_circ_0003972
ENSG00000185404	SP140L	SP140 nuclear body pro	hsa_circ_0008382
ENSG00000271699,EN	SNX29P2,AC0	sorting nexin 29 pseudo	hsa_circ_0038891
ENSG00000144118	RALB	RAS like proto-oncogen	hsa_circ_0056285
ENSG00000172340	SUCLG2	succinate-CoA ligase G	hsa_circ_0003060
ENSG00000169398	PTK2	protein tyrosine kinase	hsa_circ_0008345

Table S2 Differentially expressed miRNAs in RA by RNA-seq analysis

ID	Log2FoldChange	P.value
10_18564	2.67	3.78E-05
11_19908	1.67	0.016169263
11_20863	1.66	0.011905993
1_1655	1.56	0.027946021
13_23379	1.45	0.033050499
14_25108	1.67	0.016169263
15_26190	2.20	0.001001147
17_27973	1.67	0.016169263
18_29671	1.49	0.040311402
19_30171	3.97	0.00125122
20_31366	1.62	0.021261083
4_9649	1.32	0.031149879
6_13619	1.56	0.016541167
8_16716	-2.35	0.041605228
hsa-miR-1180-3p	2.28	1.72E-05
hsa-miR-1294	2.24	0.000698543
hsa-miR-129-5p	2.45	0.004961222
hsa-miR-1303	2.13	0.007659642
hsa-miR-1304-5p	2.77	0.029774498
hsa-miR-144-3p	1.87	9.19E-06
hsa-miR-144-5p	2.04	0.00040654
hsa-miR-146a-3p	1.12	0.040923745
hsa-miR-151a-5p	-2.13	0.013799535
hsa-miR-16-2-3p	1.30	0.003898705
hsa-miR-181a-2-3p	-1.86	0.037451457
hsa-miR-182-5p	2.58	1.09E-08
hsa-miR-183-5p	4.36	1.88E-18
hsa-miR-18a-3p	2.01	0.007985414
hsa-miR-219a-1-3p	1.87	0.039607375
hsa-miR-29a-3p	-1.80	0.045221014
hsa-miR-29b-3p	-1.86	0.03313504
hsa-miR-29c-3p	-2.25	0.021617613
hsa-miR-30b-5p	-1.94	0.01518803
hsa-miR-30e-5p	-2.16	0.007124937
hsa-miR-3120-5p	1.57	0.004374711
hsa-miR-3127-3p	4.81	0.012216265
hsa-miR-3149	3.07	0.007673926
hsa-miR-3158-3p	2.35	1.88E-07
hsa-miR-31-5p	-2.95	0.024602066
hsa-miR-3180-5p	1.32	0.041567215
hsa-miR-3184-5p	1.07	0.012435835
hsa-miR-342-3p	-2.61	0.001096137
hsa-miR-3615	1.07	0.017365314
hsa-miR-409-5p	-2.96	0.04387379
hsa-miR-424-3p	2.61	1.98E-06
hsa-miR-4443	1.98	6.49E-06
hsa-miR-4497	2.04	0.047384053

hsa-miR-4508	3.32	0.008075989
hsa-miR-4732-5p	1.20	0.016169263
hsa-miR-4772-3p	-3.38	0.007373098
hsa-miR-486-3p	2.03	0.000122028
hsa-miR-486-5p	2.66	3.80E-09
hsa-miR-5010-3p	1.39	0.011887135
hsa-miR-5189-5p	2.43	0.01217424
hsa-miR-550a-5p	2.71	8.95E-05
hsa-miR-584-3p	3.32	0.037800303
hsa-miR-597-3p	1.26	0.040693893
hsa-miR-6503-3p	3.70	0.018863784
hsa-miR-7848-3p	1.19	0.038707293
hsa-miR-7976	4.27	0.000307902
hsa-miR-92b-3p	1.39	0.007159679
hsa-miR-92b-5p	5.81	8.78E-05
hsa-miR-96-5p	5.03	1.78E-18
